# Factors Associated with Treatment Burden in Older Adults with Multimorbidity and Implications for Potential Reduction Strategies: A Scoping Review

**DOI:** 10.3390/healthcare14172670

**Published:** 2026-08-22

**Authors:** Leandro Amato, Gianluca Ciccaglione, Noemi Giannetta, Emanuele Di Simone, Nicolò Panattoni, Sara Dionisi, Alessandra Improta, Sofia Taborri, Marco Di Muzio

**Affiliations:** 1 Department of Biomedicine and Prevention, University of Rome Tor Vergata, 00133 Rome, Italy; leandro.amato@uniroma1.it (L.A.); improtalessandra@gmail.com (A.I.); 2 Department of Clinical and Molecular Medicine, Sapienza University of Rome, 00185 Rome, Italy; gianluca.ciccaglione@virgilio.it; 3 Departmental Faculty of Medicine, Saint Camillus International University of Health and Medical Sciences (UniCamillus), 00131 Rome, Italy; noemi.giannetta@unicamillus.org; 4 Department of Medical, Movement and Wellbeing Sciences, Parthenope University of Naples, 80133 Naples, Italy; emanuele.disimone@uniparthenope.it; 5 Nursing Research Unit IFO, IRCCS Regina Elena National Cancer Institute, 00144 Rome, Italy; nicolo.panattoni@ifo.it; 6 Nursing, Technical, Rehabilitation Department, DaTeR Azienda Unità Sanitaria Locale di Bologna, 40124 Bologna, Italy; sara.dionisi2@unibo.it; 7 Department of Wellbeing, Health and Environmental Sustainability, Sapienza University of Rome, 00185 Rome, Italy; marco.dimuzio@uniroma1.it

**Keywords:** older adults, treatment burden, multimorbidity, deprescribing, medication adherence, scoping review

## Abstract

Background: Treatment Burden (TB) refers to the workload imposed on patients by healthcare-related tasks involved in the management of chronic conditions. Despite increasing evidence on this phenomenon, strategies for its reduction in older adults with multimorbidity remain poorly explored. Aim: To map the factors associated with treatment burden reported for older patients with multimorbidity and to derive implications for potential reduction strategies, regardless of the care setting. Methods: A scoping review was conducted applying the JBI methodology for scoping reviews and reported in accordance with the PRISMA-ScR checklist. The literature search was performed on PubMed, Scopus, and CINAHL; no restrictions on publication year or language were applied. All study designs were eligible if they reported factors associated with TB from which reduction strategies could be derived, in patients aged ≥65 years, or with a mean age ≥ 65 years, with multimorbidity or associated geriatric conditions. Results: Six studies met the eligibility criteria, all of which were observational in design, conducted between 2021 and 2025. Eleven potential strategies for TB reduction, inferred from observational findings, were identified and grouped into three areas: healthcare system and access to care, therapeutic regimen, and patient and psychosocial context. Conclusions: To our knowledge, this review provides the first structured mapping of potential TB reduction strategies specifically in the older population with multimorbidity. The identified strategies, derived by the authors rather than empirically tested, may exclusively inform the design of future interventional studies and offer healthcare professionals preliminary guidance for TB management in this population.

## 1. Introduction

Over the past fifteen years, treatment burden (TB) has received growing attention in the literature. It describes the workload imposed on patients and their caregivers by the sum of healthcare, logistical, and management activities required to cope with chronic conditions [1]. This concept is distinct from the traditional *burden of illness*, which encompasses only the direct effects of disease, such as pain, disability, and mortality, irrespective of the demands generated by its treatment [2]. TB is a multidimensional phenomenon, encompassing several domains: medication regimens, medical appointments, health monitoring, administrative tasks, lifestyle adjustments, and social challenges [3]. Its intensity is influenced by the healthcare system, socioeconomic status, and level of education [3]. The burden is therefore patient-specific: two individuals with similar clinical profiles and therapeutic pathways may experience extremely different levels of burden [4]. Although a number of assessment instruments have been developed to measure this phenomenon [5,6,7], their systematic adoption in clinical practice remains limited [8].

Many therapeutic tasks, taken individually, may seem easy to handle. However, their cumulative effect can overwhelm patients and caregivers [4]. This effect is compounded when multiple chronic conditions coexist: adhering to simultaneous treatment programmes not only multiplies the demands of individual activities, but generates additional burdens related to coordination across specialists, management of conflicts between medication regimens, and fragmentation of care [4,9]. Multimorbidity, defined as the co-occurrence of two or more chronic conditions [10], is particularly prevalent among older adults (≥65 years): a recent meta-analysis of 126 studies across 54 countries reported a global prevalence of 37.2% among adults, rising to 51.0% among those aged 60 years and older [11], who represent a growing proportion of the global population [12]. It is further associated with higher mortality risk [13] and increased risk of hospitalisation [14]. No single condition is treated as primary in multimorbidity, unlike in comorbidity, which is anchored to a primary index disease [15]. Morbidity, by contrast, refers simply to the presence of any pathological condition, with no implication of multiple co-occurring conditions at all [16]. Older adults, particularly exposed to this phenomenon, frequently present with functional limitations, cognitive impairment, declining socioeconomic resources, and social isolation, and independently face a greater risk of further physical function decline over time [17,18,19]. These factors reduce their capacity to cope with the demands of therapeutic programmes, placing them at higher risk of TB [4,8,9]. High TB levels are associated with several adverse outcomes: reduced medication adherence (MA), deterioration in quality of life, increased hospital admissions and mortality, higher healthcare costs and greater caregiver burden [20,21]. Among these, the inversely proportional relationship between TB and MA is particularly relevant: as TB increases, adherence declines [22]. Difficulty in following treatment regimens leads to clinical deterioration, which generates even more complex therapeutic demands [9].

The *Minimally Disruptive Medicine* (MDM) framework proposes to address this problem by reducing the therapeutic workload placed on patients while strengthening their capacity to manage it, through care that is coordinated around the patient’s needs [4,23,24]. Workload refers to the cumulative demands imposed on the patient by disease and its treatment. Capacity, by contrast, encompasses the resources a patient can mobilise to meet those demands: physical and cognitive function, psychological resilience, financial resources, and social support. Consistent with Shippee et al.’s Cumulative Complexity model, treatment burden emerges when workload exceeds capacity, and the two are dynamically linked: a rise in workload can itself deplete capacity, and a loss of capacity can make an unchanged workload feel heavier [9]. Despite growing attention to this phenomenon, strategies for reducing TB in older adults with multimorbidity remain poorly explored [8]. Most of the available literature has focused on describing and measuring TB rather than on identifying ways to reduce it [8]. Lee et al. recently conducted an integrative review on TB in multimorbidity [8], but did not specifically map reduction strategies for the older population, which has a distinct vulnerability profile compared to the general adult population [17,18].

This scoping review aims to map the factors associated with treatment burden in patients aged ≥65 years with multimorbidity, as reported in the available literature, and to identify implications for potential reduction strategies, regardless of the care setting. Specifically, the factors identified, the instruments used to measure treatment burden, and the outcomes assessed will be described, with a view to informing future interventional research.

## 2. Materials and Methods

A scoping review was conducted between January and March 2026 to identify and classify potential strategies for TB reduction in older patients with multimorbidity. The review was conducted in accordance with the JBI methodology for scoping reviews [25] and reported following the PRISMA-ScR guidelines [26]; the completed checklist is provided as Supplementary File S1. The protocol was registered on the Open Science Framework (OSF) prior to manuscript submission (OSF: https://doi.org/10.17605/OSF.IO/JW9ED). The research question, eligibility criteria, databases, and search strings were defined before the literature search was conducted. The thematic grouping of identified strategies was developed inductively during data charting: one reviewer proposed the grouping based on conceptual similarity among the strategies derived from the included studies’ findings, and all co-authors subsequently discussed and approved it. This process did not constitute a formal reflexive thematic analysis. The material grouped consisted of statements already formulated as discrete strategies; no interpretive coding of raw data was involved. The research question guiding the review was “What factors associated with treatment burden are described in the literature for older patients (≥65 years) with multimorbidity, and what implications for reduction strategies can be derived from the available evidence, regardless of the care setting?” (PCC framework—Population: older patients (≥65 years) with multimorbidity or associated geriatric conditions including frailty, functional disability, or geriatric syndromes, documented by study authors as a clinically relevant characteristic of the population; Concept: factors associated with TB and their implications for potential reduction strategies; Context: any care setting).

### 2.1. Search Strategy

The search strategy was developed by the research team in collaboration with an expert librarian. Three bibliographic databases were searched: PubMed, Scopus, and CINAHL (Cumulative Index to Nursing and Allied Health Literature). These databases were selected to ensure adequate coverage of the review objectives, as they span the biomedical (PubMed), multidisciplinary (Scopus), and nursing literature (CINAHL). Grey literature was not searched, as the present study focuses on strategies derived from peer-reviewed studies. This choice is acknowledged as a limitation of the study (see Limitations). Keywords were combined using the Boolean operators “OR” and “AND”, constructing search strings adapted for each database (Table 1). No filters were applied regarding publication year or language.

### 2.2. Study Selection

Inclusion and exclusion criteria were established a priori. All study designs were considered eligible (experimental, observational, systematic reviews, qualitative and mixed-methods studies). Studies were included if they reported factors associated with TB from which reduction strategies could be derived, in patients aged ≥65 years with multimorbidity or associated geriatric conditions, such as frailty, functional disability, or geriatric syndromes, documented as a relevant characteristic of the population by the authors. In the absence of an explicit age cut-off, studies were included when the examined population had a mean age ≥ 65 years or when results were stratified by age groups. This selection criterion was introduced to avoid excluding relevant evidence from studies conducted on broader adult populations but with findings applicable to the older age group. Conversely, studies were excluded if they reported TB purely as a descriptive or measurement outcome, with no associated factors from which implications for reduction could be derived. Studies were also excluded if they were conducted in populations with a mean age below 65 years or without age-group stratification; if multimorbidity or associated geriatric conditions were not documented as a relevant characteristic of the population; and if published exclusively as abstracts, editorials, letters to the editor, or opinion pieces.

**Figure 1:**
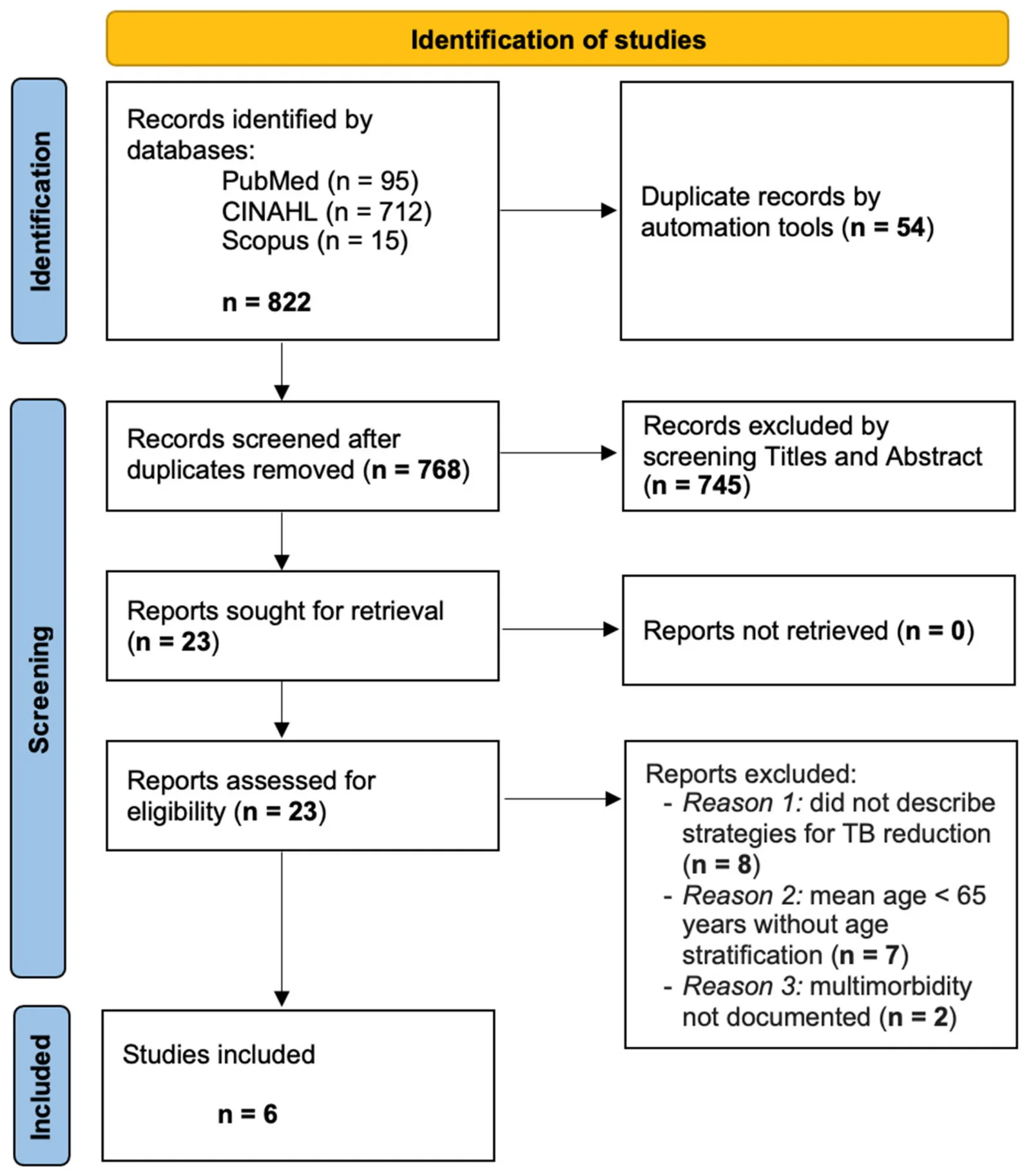
PRISMA-ScR flow diagram.

Selection proceeded in two stages: the first screening by title and abstract, followed by full-text review of potentially eligible studies. Both stages were conducted independently by two reviewers; disagreements were resolved through discussion. Formal inter-rater agreement (Cohen’s kappa) was not calculated. Zotero^®^ (version 7) bibliographic software was used as a citation manager for all the retrieved articles. Duplicates were removed automatically using the software’s built-in function and subsequently verified manually by the reviewers. The selection process is illustrated in the PRISMA flow diagram (Figure 1). Consistent with JBI methodology for scoping reviews, formal critical appraisal was not performed, as scoping reviews aim to map the extent of available evidence rather than assess its quality [25].

### 2.3. Data Extraction

Data extraction was conducted independently by two reviewers. A data charting table was used to synthesise the extracted data. The following information was recorded for each included study: author(s), year of publication, country, study design, objective(s), study population, instruments used, and care setting (Table 2).

## 3. Results

The literature search retrieved 822 records. After removal of 54 duplicates, 768 were screened by title and abstract. Of these, 745 were excluded and 23 full texts were assessed for eligibility. At the end of the selection process, 6 studies met the eligibility criteria and were included in the review (Figure 1).

### 3.1. Study Characteristics

All included studies were published between 2021 and 2025. All were observational in design: four cross-sectional [27,28,29,30], one of which was multicentre [29], and two longitudinal [31,32], one of which was retrospective [32]. The studies were conducted in five countries: two in the United States [30,32], one in the United Kingdom [31], one in Ethiopia [29], one in Turkey [27], and one in China [28].

All studies were conducted in populations of older patients with multimorbidity. One study included patients with COPD with a high prevalence of multimorbidity [27]. Another examined a large cohort of patients with a new diagnosis of non-muscle-invasive bladder cancer, in whom multimorbidity and associated geriatric conditions were documented as a clinically relevant characteristic of the population [32].

Sample sizes were heterogeneous: five studies included between 300 and 1795 patients [27,28,29,30,31], while the retrospective study by Garg et al. analysed a cohort of 73,395 patients [32]. Three studies were conducted in outpatient and/or hospital settings [27,29,32], two in community settings [28,30], and one in primary care [31]. Three of the six included studies enrolled participants below the age of 65 years [28,30,31] while one did not specify an age eligibility criterion but had a mean age of 67.7 years [27].

#### Measurement Tools

The included studies employed heterogeneous measures to evaluate TB. Five of the six studies used validated patient-reported instruments: three adopted the Multimorbidity Treatment Burden Questionnaire (MTBQ) [5], in versions translated and adapted to the study population [28,29,31], and two used the Treatment Burden Questionnaire (TBQ) [6], an instrument more appropriate when a single predominant condition is present [27,30]. Garg et al. instead used an indirect measure of TB based on the number of healthcare contacts and the intensity of procedures in the 12 months following diagnosis [32]. This measure reflects only the logistical and administrative dimension of the construct, without accounting for patients’ subjective perception of burden.

### 3.2. Strategies

The following potential strategies were not tested as interventions in any included study. They represent implications derived by the present authors from the observational findings of the included studies, informed by the factors identified as significantly associated with treatment burden. Eleven potential strategies were identified and grouped into three areas: healthcare system and access to care [29,30,31,32], therapeutic regimen [27,29,32], and patient and psychosocial context [27,28,29,30] (Table 3). This grouping was proposed by one author based on conceptual similarity and approved by all co-authors. The same strategies were also classified by mechanism according to the workload/capacity framework of the Cumulative Complexity Model [9] (Table 4).

Within the healthcare system area, Hounkpatin et al. report that living more than ten minutes from general practice services is associated with an increase in TB over time and suggest the need for interventions to improve the proximity and accessibility of primary care services [31]. This strategy is indicated particularly for patients with limited health literacy, in whom this association is more pronounced [31]. In the context of proximity to care services, two studies found significantly higher non-adherence rates [29] and a greater number of healthcare contact days among patients living in rural compared to urban areas [32]. The authors identify, among the reduction strategies, the strengthening of social support, health education, and care networks in rural areas [29]. Zhang et al. and Garg et al. suggest the use of telehealth or home-based care as strategies to reduce the logistical burden in patients for whom healthcare visits represent the primary source of burden, particularly in rural areas [30,32]. They also call for structural reforms to reduce the administrative and financial burden, which emerges as one of the main sources of TB reported in the United States population [30].

In the context of the therapeutic regimen, Sönmez & Top highlight reduction in the number of medications and simplification of the therapeutic regimen as strategies to alleviate TB and improve medication adherence [27]; Dagnew et al. similarly call for simplified regimens [29]. Treatment complexity, in terms of pharmaceutical formulations and additional instructions, was found to be an independent predictor of TB [27]. The authors emphasised the need to incorporate TB assessment and monitoring into clinical guidelines for the management of chronic diseases [27]. In the oncological setting, Garg et al., citing evidence on shared decision-making and overtreatment, suggest that aligning treatment with patient goals and preferences may help reduce the treatment burden associated with overtreatment in older adults with cancer [32]. At the patient and psychosocial level, Sönmez & Top report that social support and self-efficacy are predictors of TB reduction [27]. They cite evidence that social support protects against TB by reducing health-related concerns, offering financial assistance, and easing stress. Based on this, they recommend organising educational programmes to strengthen patients’ self-efficacy [27]. Reduced self-efficacy and the presence of anxiety and depression were found to be associated with higher levels of TB and lower motivation for health management behaviours [28]. This association points to the treatment of psychological distress as a potential strategy for containing TB. Zhang et al. suggest identifying the factors that limit patients’ capacity to cope with daily healthcare demands, including functional, sensory, and psychological deficits, and directing interventions towards strengthening these capacities [30]. Finally, Dagnew et al. report that caregiver-managed therapy is associated with a higher likelihood of non-adherence compared to self-management, highlighting the value of strengthening self-management capacities in older patients [29].

**Figure 2:**
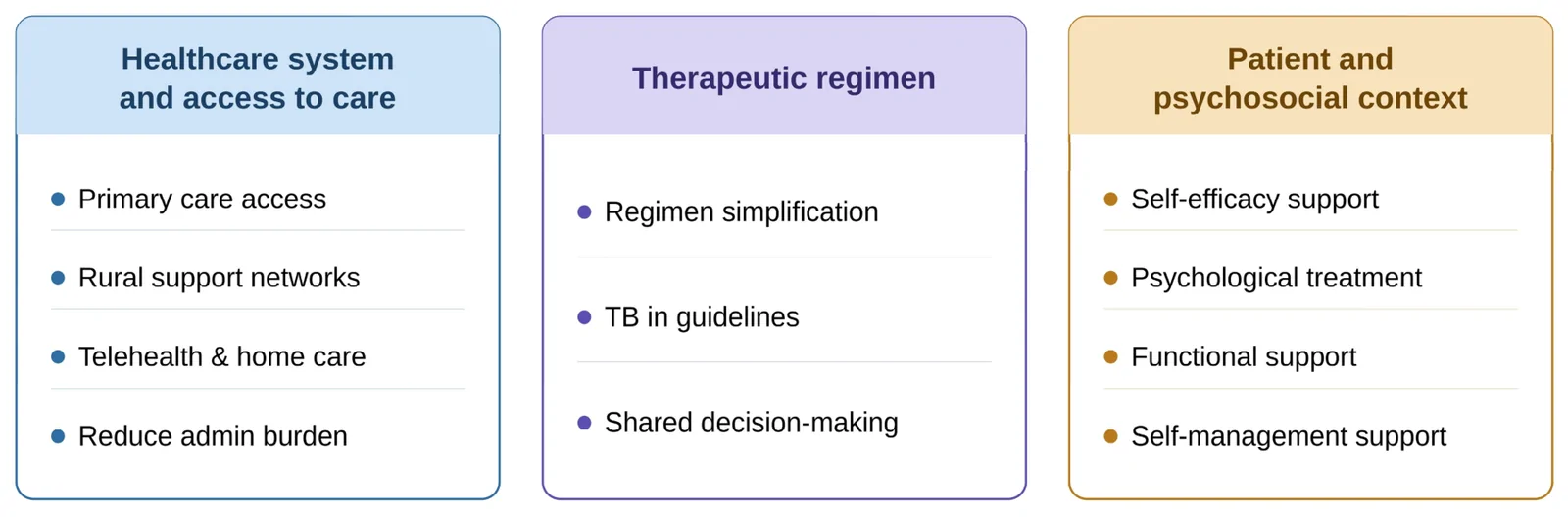
Visual summary of the strategies detailed in Table 3, with abbreviated labels.

Figure 2 shows a visual summary of these strategies, with abbreviated labels.

## 4. Discussion

This scoping review represents, to our knowledge, the first attempt to systematically map potential TB reduction strategies specifically in the older population with multimorbidity. Six observational studies published between 2021 and 2025 [27,28,29,30,31,32] yielded eleven potential strategies, grouped into three areas: healthcare system and access to care, therapeutic regimen, and patient and psychosocial context (Figure 2). These are consistent with the approach proposed by the Minimally Disruptive Medicine framework, which aims to reduce the therapeutic workload imposed on patients while strengthening their capacity to manage it [23]. These strategies, however, are derived from observational studies and should not be interpreted as evidence of intervention effectiveness. Research on TB in this population has so far focused on describing and measuring it rather than on testing ways to reduce it. The term ‘strategy’ is therefore used throughout in a hypothetical sense.

A first element to consider concerns the systemic nature of the access-to-care problem. The three studies documenting the association between distance from care services and increased TB [31,32] or reduced medication adherence [29] show that TB is not simply a reflection of geographical disparity, but is partly shaped by healthcare system organisation [33]. The only prospective longitudinal study in this review, Hounkpatin et al. [31], shows that TB can worsen substantially over time in older adults with multimorbidity, with a subset of patients reaching the highest burden category directly. Distance from primary care was independently associated with this increase, particularly in patients with limited health literacy [31]. This suggests that access barriers and individual resources may both contribute to the burden patients experience over time. Healthcare systems should on the one hand intervene to facilitate physical access to care by strengthening community networks and reducing distances and waiting times [34], and on the other hand address patients’ own resources [35]. Telemedicine has been proposed as an integrated platform for support and monitoring capable of reducing physical travel [36,37], and only two included studies suggest its adoption as a strategy to reduce the logistical burden of healthcare visits [30,32]. Its use in older adults should nonetheless be carefully evaluated, as remote consultations may be perceived as a reduction in contact with healthcare professionals and consequently increase TB levels [38,39]. Digital health tools may add to rather than reduce patients’ burden [38]. Many older adults lack internet access or the skills to navigate digital platforms independently [38,40,41]. For patients with multimorbidity, operating multiple separate telemonitoring systems can be overwhelming [38]. Platforms that are difficult to use may leave patients feeling abandoned by their care [38,41]. This is consistent with recent systematic reviews on digital interventions for treatment burden in chronic conditions: digital tools can alleviate burden, but in patients with low digital literacy they may increase it, depending on design and support level [42,43]. This ambivalence could be addressed at the intervention design stage, by actively involving older patients and supporting digital literacy and family involvement [38,40,41]. Beyond physical distance, the financial cost of care is an equally relevant barrier to access [30], as highlighted by the MDM framework [23]. Although the mechanisms of financial burden differ between younger and older adults [44], for the latter the reduction in retirement income, the higher frequency of healthcare contacts, and the cumulative costs of polypharmacy create a specific vulnerability profile [45].

In reducing pharmacological burden, quantitative deprescribing [24] should be accompanied by qualitative simplification of the therapeutic regimen, as the latter can significantly affect overall burden [25,29]. Deprescribing safely reduces the number of medication and, in several trials, improves quality of life or reduces hospitalisation in older adults with polypharmacy [46]. Reviewing treatment plans and prescribing therapies compatible with patients’ schedules and personal habits would allow older adults more free time to carry out their daily activities [21,47]. Individual negotiation of care goals allows therapeutic management to be approached from a shared perspective, balancing clinical objectives and perceived burden [32], and may result in a personalised treatment plan that positively influences MA [22]. Multifactorial interventions incorporating shared goal setting may improve patient-centred care outcomes in older patients with multimorbidity, though evidence remains limited to a small number of controlled trials [48].

Self-efficacy can reduce TB, but it is also a resource that TB itself erodes [27,28]. This dynamic is consistent with Shippee et al.’s Cumulative Complexity model, which describes how workload and patient capacity mutually influence each other [9]. Reducing the burden frees up patient resources; strengthening those resources makes the patient more capable of sustaining the burden [27]. Isolated interventions targeting a single area may therefore have limited effectiveness [9]. Mapping the eleven strategies onto the workload/capacity framework (Table 4) suggests that the three areas identified in Table 3 broadly align with this theoretical distinction. Strategies concerning the healthcare system and the therapeutic regimen appear to act predominantly by reducing workload, whereas those concerning the patient and psychosocial context predominantly enhance capacity. TB assessment integration into clinical guidelines is the exception: rather than directly reducing workload or enhancing capacity, it may instead help identify which patients face an imbalance between the two, and which lever is most relevant for them. In our view, TB reduction requires an integrated approach targeting the system, the regimen, and the patient’s resources at once. Social support, in this perspective, does not substitute the patient in care management [27,29], but could strengthen self-management competencies [27].

Finally, given the lack of experimental evidence on specific interventions, we consider that systematic assessment of psychological status should be part of the clinical evaluation of TB. In our view, TB measurement represents the prerequisite for any reduction strategy. Five of the six included studies used validated instruments such as the MTBQ [5] and the TBQ [6], yet their adoption in routine clinical practice remains limited [8]. Only one included study emphasises the need to incorporate TB assessment into clinical guidelines [27], yet none describes a protocol integrated into standard care pathways. As a result, patients who might benefit from targeted strategies may go unidentified. The included studies were also conducted in markedly different healthcare contexts. Despite this heterogeneity, pharmacological burden and social support appeared as recurring themes, suggesting these may be relevant dimensions of TB in different settings.

Measurement heterogeneity across the included studies may have shaped not only the magnitude of reported associations but which strategies could emerge at all. TBQ and MTBQ both cover medication, appointments, self-monitoring, and lifestyle, though item content varies somewhat across specific versions of each instrument. TBQ is a general measure validated across conditions [27,30], while MTBQ was developed specifically for multimorbidity [28,29,31], and the two capture the construct through a different population lens. Garg et al., by contrast, measured only objective contact days with the health system, with no subjective perception of burden [32]. This distinction matters concretely: self-efficacy support [27] and psychological distress treatment [28] came only from the two studies where patients answered questions directly. Garg et al.’s administrative count could not have produced either strategy, simply because the instrument had no way to register it.

Beyond this, some strategies rest on firmer ground than others. Telehealth and home-based care [30,32] and regimen simplification [27,29] each appeared in two studies that used different instruments, so they are unlikely to be an artefact of any one instrument. By contrast, shared decision-making [32], self-efficacy support [27], and psychological distress treatment [28] each come from a single instrument only, and whether they would hold up under a different one is still unknown.

### Limitations

This review has several limitations. The most significant is the exclusively observational nature of the included studies. In the absence of experimental designs, causal relationships could not be established, and the identified strategies cannot be translated into operational recommendations. The heterogeneity across studies in terms of population, setting, and TB measurement instruments further limits direct comparison of findings. MTBQ, TBQ, and Garg et al.’s healthcare-utilizasion proxy captures different constructs of TB, introducing a degree of interpretative heterogeneity. Although the mean age criterion was established a priori to broaden the evidence base in a topic with limited literature, some included studies enrolled participants below 65 years [28,30,31], which may limit the direct applicability of the identified strategies to the strictly older population. Inter-rater agreement was not formally quantified through Cohen’s kappa, which is an additional limitation. The literature search, confined to three databases, may not have identified all relevant studies. In addition, the absence of grey literature searching and hand searching of relevant journals is a known methodological limitation in scoping reviews [25], which may have introduced a bias in favour of positive peer-reviewed literature. Finally, the conduct of studies across diverse economic contexts limits the transferability of conclusions to settings with substantially different resources and healthcare structures.

Future research should prioritise randomised controlled trials and quasi-experimental designs that can directly test the effectiveness of the identified strategies in older adults with multimorbidity. The adoption of instruments specifically developed for multimorbidity contexts, such as the MTBQ [5], would improve comparability across studies. Extending research to low- and middle-income countries would broaden the evidence base and improve the global applicability of the identified strategies.

## 5. Conclusions

To our knowledge and based on the databases searched, this review provides the first structured mapping of factors associated with treatment burden in older patients with multimorbidity, along with their implications for potential reduction strategies. The potential strategies, derived from observational evidence, are grouped into three areas: healthcare system and access to care, therapeutic regimen, and patient and psychosocial context. These represent author-derived conceptual implications rather than empirically validated interventions. None have been tested in practice, so their real-world effectiveness is unknown. In the absence of experimental studies, they may exclusively inform the design of future interventional research and offer healthcare professionals preliminary guidance on TB management in this population. In primary care and outpatient settings, routine TB assessment with validated instruments may represent a useful first step to identify patients at risk, though its integration into standard clinical care has not itself been tested and its impact on patient outcomes remains to be established.

## Figures and Tables

**Table 1:** Search strings.

Database	String
PubMed	(“treatment burden”[Title/Abstract] OR “burden of treatment”[Title/Abstract] OR “treatment load”[Title/Abstract] OR “medication burden”[Title/Abstract]) AND (intervention*[Title/Abstract] OR program*[Title/Abstract] OR strateg*[Title/Abstract] OR management[Title/Abstract] OR trial[Title/Abstract]) AND (older adult*[Title/Abstract] OR elderly[Title/Abstract] OR geriatric*[Title/Abstract] OR aged[Title/Abstract] OR senior*[Title/Abstract] OR “older people”[Title/Abstract]) AND (multimorbidity[Title/Abstract] OR multi-morbidity[Title/Abstract] OR “multiple chronic conditions”[Title/Abstract] OR comorbidity[Title/Abstract] OR chronic disease*[Title/Abstract])
Scopus	TITLE-ABS (treatment burden OR burden of treatment OR treatment load OR medication burden) AND TITLE-ABS (intervention* OR program* OR strateg* OR management OR trial) AND TITLE-ABS (older adult* OR elderly OR geriatric* OR aged OR senior* OR older people) AND TITLE-ABS (multimorbidity OR multi-morbidity OR multiple chronic conditions OR comorbidity OR chronic disease*)
CINAHL (via EBSCO)	XB (treatment burden OR burden of treatment OR treatment load OR medication burden) AND XB (intervention* OR program* OR strateg* OR management OR trial) AND XB (older adult* OR elderly OR geriatric* OR aged OR senior* OR older people) AND XB (multimorbidity OR multi-morbidity OR multiple chronic conditions OR comorbidity OR chronic disease*)

**Table 2:** Data extraction table.

Author(s)—Year—Country	Study Design	Objective(s)	Study Population	Instruments	Care Setting
Sönmez S. & Top M.—2025—Turkey [27]	Cross-sectional study	To identify the factors associated with TB in older adults and its mediating role between comorbidity and quality of life.	332 patients diagnosed with COPD and high prevalence of multimorbidity. Mean age = 67.7 years. Eligibility: not specified.	Treatment Burden Questionnaire (TBQ); COPD Assessment Test (CAT); Comorbidity and Oncological Treatment Evaluation (COTE) Index; Medication Regimen Complexity Index (MRCI); Multidimensional Scale of Perceived Social Support (MSPSS); Self-Efficacy for Managing Chronic Disease scale (SEMCD).	Outpatient department
Guo Y. et al.—2025—China [28]	Cross-sectional study	To evaluate the risk factors for TB in Chinese older adults with multimorbidity and its relationship with self-efficacy.	1599 patients with multimorbidity. Mean age = 71.5 years. Eligibility: ≥60 years.	Multimorbidity Treatment Burden Questionnaire (MTBQ); Self-Efficacy for Managing Chronic Disease 6-item scale (SEMCD6)	Community
Dagnew S.B. et al.—2025—Ethiopia [29]	Multicentre cross-sectional study	To evaluate treatment burden and medication adherence in older adults in specialized hospitals in the Amhara region.	422 patients with multimorbidity and polypharmacy. Mean age = 71.8 years. Eligibility: ≥65 years.	Multimorbidity Treatment Burden Questionnaire (MTBQ); Charlson Comorbidity Index (CCI); Medication Regimen Complexity Index (MRCI); General Medication Adherence Scale (GMAS)	Outpatient department/hospital
Zhang A.D. et al.—2025—United States [30]	Cross-sectional study	To quantify TB in adults ≥ 50 years, compare it between the 50–64 and ≥65 years age groups, and analyse its association with sociodemographic, health, and functional characteristics.	1795 patients with multimorbidity. Mean age = 68.5 years. Eligibility: ≥50 years.	Treatment Burden Questionnaire (TBQ); Activities of Daily Living (ADL); Instrumental Activities of Daily Living (IADL)	Community
Hounkpatin H.O. et al.—2022—United Kingdom [31]	Longitudinal study	To measure change in TB over time, identify related factors, and evaluate a single-item measure for identifying high workloads in older adults with multimorbidity.	300 patients with multimorbidity. Mean age = 74.5 years. Eligibility: ≥55 years.	Multimorbidity Treatment Burden Questionnaire (MTBQ)	Primary care
Garg T. et al.—2021—United States [32]	Retrospective longitudinal study	To evaluate the association between geriatric conditions and TB after diagnosis of non-muscle-invasive bladder cancer in older adults.	73,395 patients newly diagnosed with non-muscle-invasive bladder cancer, with multimorbidity. Mean age = 77.5 years. Eligibility: ≥66 years.	Indirect measure of TB based on number of healthcare contacts (outpatient care, emergency department visits, and hospitalization) in the 12 months following diagnosis.	Outpatient department/hospital

**Table 3:** Proposed strategies for TB reduction *.

Area	Strategy	Reference
Healthcare system and access to care	Improved proximity and accessibility of primary care services	[31]
	Social support, health education, and care network strengthening in rural areas	[29]
	Telehealth and home-based care	[30,32]
	Structural reforms to reduce administrative and financial burden	[30]
Therapeutic regimen	Medication reduction and regimen simplification	[27,29]
	TB assessment integration into clinical guidelines	[27]
	Shared decision-making to reduce overtreatment	[32]
Patient and psychosocial context	Self-efficacy strengthening through education and financial support	[27]
	Psychological distress treatment	[28]
	Strengthening of functional, sensory, and psychological capacities	[30]
	Self-management support	[29]

* Strategies represent implications derived by the present authors from associations reported in the included studies; grouping into the three areas was proposed by one author based on conceptual similarity and approved by all co-authors.

**Table 4:** Proposed strategies mapped onto the workload/capacity framework.

Mechanism	Strategies
Reduces workload	Improved proximity and accessibility of primary care services [31]; Telehealth and home-based care [30,32]; Structural reforms to reduce administrative and financial burden [30]; Medication reduction and regimen simplification [27,29]; Shared decision-making to reduce overtreatment [32]
Enhances capacity	Social support, health education, and care network strengthening in rural areas [29]; Self-efficacy strengthening through education and financial support [27]; Psychological distress treatment [28]; Strengthening of functional, sensory, and psychological capacities [30]; Self-management support [29]
Enables identification of imbalance	TB assessment integration into clinical guidelines [27]

## Data Availability

No new data were created or analyzed in this study. Data sharing is not applicable to this article.

## Supplementary Materials

The following supporting information can be downloaded at: https://www.mdpi.com/article/10.3390/healthcare14172670/s1, Preferred Reporting Items for Systematic reviews and Meta-Analyses extension for Scoping Reviews (PRISMA-ScR) Checklist.

## Abbreviations

The following abbreviations are used in this manuscript:

TB	Treatment Burden
MA	Medication Adherence
JBI	Joanna Briggs Institute
PRISMA-ScR	Preferred Reporting Items for Systematic reviews and Meta-Analyses extension for Scoping Reviews
MDM	Minimally Disruptive Medicine
OSF	Open Science Framework
PCC	Population, Concept, Context
CINAHL	Cumulative Index to Nursing and Allied Health Literature
COPD	Chronic Obstructive Pulmonary Disease
MTBQ	Multimorbidity Treatment Burden Questionnaire
TBQ	Treatment Burden Questionnaire
